# Comparison of Expression Profiles in Ovarian Epithelium *In Vivo* and Ovarian Cancer Identifies Novel Candidate Genes Involved in Disease Pathogenesis

**DOI:** 10.1371/journal.pone.0017617

**Published:** 2011-03-15

**Authors:** Catherine Emmanuel, Natalie Gava, Catherine Kennedy, Rosemary L. Balleine, Raghwa Sharma, Gerard Wain, Alison Brand, Russell Hogg, Dariush Etemadmoghadam, Joshy George, Australian Ovarian Cancer Study Group, Michael J. Birrer, Christine L. Clarke, Georgia Chenevix-Trench, David D. L. Bowtell, Paul R. Harnett, Anna deFazio

**Affiliations:** 1 Department of Gynaecological Oncology, Westmead Hospital, Westmead, New South Wales, Australia; 2 Westmead Institute for Cancer Research, Westmead Millennium Institute, Westmead Hospital and University of Sydney, Westmead, New South Wales, Australia; 3 Department of Anatomical Pathology, Westmead Hospital, Westmead, New South Wales, Australia; 4 Cancer Genomics and Genetics, Peter MacCallum Cancer Centre, Melbourne, Victoria, Australia; 5 Cancer Genetics Laboratory, Division of Genetics and Population Health, Queensland Institute of Medical Research, Brisbane, Queensland, Australia; 6 Department of Biochemistry and Molecular Biology, University of Melbourne, Melbourne, Victoria, Australia; 7 Department of Medicine, Harvard Medical School, Boston, Massachusetts, United States of America; Victor Chang Cardiac Research Institute (VCCRI), Australia

## Abstract

Molecular events leading to epithelial ovarian cancer are poorly understood but ovulatory hormones and a high number of life-time ovulations with concomitant proliferation, apoptosis, and inflammation, increases risk. We identified genes that are regulated during the estrous cycle in murine ovarian surface epithelium and analysed these profiles to identify genes dysregulated in human ovarian cancer, using publically available datasets. We identified 338 genes that are regulated in murine ovarian surface epithelium during the estrous cycle and dysregulated in ovarian cancer. Six of seven candidates selected for immunohistochemical validation were expressed in serous ovarian cancer, inclusion cysts, ovarian surface epithelium and in fallopian tube epithelium. Most were overexpressed in ovarian cancer compared with ovarian surface epithelium and/or inclusion cysts (EpCAM, EZH2, BIRC5) although BIRC5 and EZH2 were expressed as highly in fallopian tube epithelium as in ovarian cancer. We prioritised the 338 genes for those likely to be important for ovarian cancer development by *in silico* analyses of copy number aberration and mutation using publically available datasets and identified genes with established roles in ovarian cancer as well as novel genes for which we have evidence for involvement in ovarian cancer. Chromosome segregation emerged as an important process in which genes from our list of 338 were over-represented including two (*BUB1*, *NCAPD2*) for which there is evidence of amplification and mutation. NUAK2, upregulated in ovarian surface epithelium in proestrus and predicted to have a driver mutation in ovarian cancer, was examined in a larger cohort of serous ovarian cancer where patients with lower NUAK2 expression had shorter overall survival. In conclusion, defining genes that are activated in normal epithelium in the course of ovulation that are also dysregulated in cancer has identified a number of pathways and novel candidate genes that may contribute to the development of ovarian cancer.

## Introduction

Epithelial ovarian cancer is the fifth most common cause of cancer death in women in the Western world and the leading cause of death from gynaecological malignancies. Despite the magnitude of this clinical problem, little is known about the mechanism of neoplastic transformation. Currently, insight into the pathogenesis of ovarian cancer comes from known factors that increase risk. These include inherited mutations in the *BRCA1/2* genes in a minority of cases, and a range of hormone and/or reproduction related factors more generally [1], [2]. In the latter case, hormone replacement therapy and a high cumulative number of life-time ovulations with few episodes of anovulation due to pregnancy, oral contraceptive use or breast feeding have been associated with increased risk. Conversely, ovarian cancer risk is reduced by more live-births, long-term breast feeding and oral contraceptive use [3].

The biological basis for altered risk associated with hormonal and reproductive factors is essentially unknown, although several hypotheses have been proposed. The first, the ‘incessant ovulation hypothesis’, posits that ovulation and its sequelae increases the likelihood of malignancy [1], and that pregnancies and oral contraceptives are protective because they suppress ovulation [4]. The second hypothesis is that circulating levels of gonadotropins increase the risk of malignancy and that pregnancy and oral contraceptive use protect by suppressing secretion of these hormones [5]. Excessive levels of gonadotropins, LH and FSH, related to the surge occurring during ovulation, are proposed to contribute to ovarian cancer development. The loss of gonadal negative feedback at menopause, resulting in peak concentrations of FSH and LH at the age when the incidence of ovarian cancer climbs dramatically, provides support for the gonadotropin hypothesis [6] and LH levels have been reported to be elevated in BRCA1 mutation carriers in the follicular phase compared with non-carriers, suggesting that high levels of LH may contribute to BRCA-associated increased risk of ovarian cancer [7]. Protection afforded by multiple pregnancies and long-term oral contraceptive use provides some support for the gonadotropins theory as both factors are associated with low levels of gonadotropins as well as the inhibition of incessant ovulation. However, the level of protection conferred by pregnancy and oral contraceptive use, has been suggested to be greater than that from inhibition of ovulation alone [2] and a third potential explanation based on epidemiological data is that the ovarian surface epithelium is protected from malignant transformation by exposure to progesterone or progesterone analogues during pregnancy or in oral contraceptives [2], [8].

Although it is widely believed that serous ovarian cancers arise from the ovarian surface epithelium and inclusion cysts formed when ovarian surface epithelium become trapped inside the ovary [9], a more recent hypothesis for the initiation of ovarian cancer suggests that precursor lesions exist in the fimbriated end of the fallopian tube epithelium [10]. It is possible that fallopian tube epithelium become trapped within the ovarian stroma during healing of the ovulatory wound where the high hormonal milieu may cause malignant transformation in a manner akin to the hypothesis for inclusion cysts [9]. Support for the initiation of ovarian cancer in fallopian tube epithelium can be found from studies which show that there are similarities in gene expression profiles of serous ovarian cancer and fallopian tube epithelium, yet these differ to the profiles observed for ovarian surface epithelium [11]. It is unclear, however, whether this is evidence of initiation in the fallopian tube epithelium or of differentiation of ovarian cancer towards a fallopian tube-like phenotype which is a defining morphological characteristic of serous ovarian cancer.

Tone et al. [11] found that gene expression profiles of fallopian tube epithelium from *BRCA* mutation carriers in the luteal phase were more similar to expression profiles of serous ovarian cancer than fallopian tube epithelium from carriers in the follicular phase. Similarly, xenograft studies have shown that xenografts of ovarian cancer are more likely to become established if they are implanted during the proestrus phase of the murine estrous cycle, when hormone levels peak [12]. These data suggest that susceptibility of ovarian surface epithelium and/or fallopian tube epithelium to malignant transformation may change throughout the estrous cycle presumably in response to fluctuating hormones, and is further evidence of a role for the menstrual cycle on ovarian cancer development.

We recently identified gene signatures associated with ovarian surface epithelium during different stages of the murine estrous cycle [13]. We reasoned that these genes which are differentially expressed in ovarian surface epithelium through the estrous cycle are likely to be hormone regulated and potentially involved in processes related to ovulation, including cell proliferation, apoptosis and inflammation. Dysregulation of pathways underpinning each of these processes has been implicated in neoplastic transformation of various tissue types. We hypothesised that a subset of genes involved in normal ovarian epithelial cell functions are also consistently aberrantly expressed in ovarian cancer and identification of this subset would assist in prioritising human candidate genes and pathways implicated in progression to ovarian cancer.

The aim of this study was to determine whether genes regulated during the estrous cycle and involved in normal ovarian function play a role in the progression of normal epithelial cells to ovarian cancer. To do this, the list of genes that was differentially expressed in ovarian surface epithelium over the estrous cycle, was cross-matched against genes with reported aberrant expression in ovarian cancer. For common genes, the expression of a number of candidates was determined by immunohistochemistry in normal ovarian surface epithelial cells, inclusion cysts, fallopian tube and ovarian cancer samples. In addition, a relationship between gene expression and copy number, and the presence of mutations in ovarian cancer was examined using existing datasets. This approach identified a number of individual candidate genes and pathways that may be involved in the pathogenesis of ovarian cancer.

## Materials and Methods

### Ethics statement

This study was approved by the Human Research Ethics Committees of Sydney West Area Health Service and the University of Sydney; protocol reference number: HREC2006/2/4.21(2293). Written informed consent was obtained from all participants in this study.

### Expression microarray analysis of murine ovarian epithelium

The expression array analysis of murine ovarian surface epithelium has previously been described in detail [13]. Briefly, total RNA (Stratagene Absolutely RNA® Nanoprep or Microprep Kit, Stratagene, La Jolla, CA), was extracted from laser microdissected ovarian surface epithelium (P.A.L.M Robot-Microbeam system, Microlaser Technologies) from BALB/c mice at 27 days of age (immature; n = 4) and 10–13 weeks of age during the estrous cycle, at proestrus (n = 4) and estrus (n = 4) [13]. Microarray slides comprising ∼15,000 expressed sequence tags from the National Institute of Ageing 15 K mouse clone library (Australian Genome Research Facility, Melbourne, Australia) were hybridized with Cy3- and Cy5-labeled cDNA generated from double-amplified RNA. Estrous stage-specific gene expression profiles were obtained by direct comparison of ovarian surface epithelium from immature mice and mice culled on proestrus evening (2200 h) and estrus morning (1000 h) [13].

### Ovarian cancer gene expression array profiles

To identify genes regulated during the estrous cycle that are expressed in human ovarian carcinoma, we compared our ovulation-related gene signature [13] with our own published gene expression profiles of ovarian cancer [14]–[16] as well as those from other selected published studies [17], [18]. We examined published studies on large-scale gene expression profiling of ovarian cancer specimens published up to August 2009 using PubMed (http://www.pubmed.com), and chose a subset of studies based on the number and histological subtype of ovarian cancer cases (serous cancer was preferred), similarity of microarray platform used and similarity of the normal reference (ovarian surface epithelium was preferred over whole ovary or cell lines). Studies which did not publish unique gene identifiers or which analysed cell lines were excluded.

All of the chosen datasets reported genes differentially expressed compared to a normal reference except for Tothill et al. [16]. We determined the genes differentially expressed in the ovarian cancer cases examined in Tothill et al. [16] by comparing the expression profiles to data from ovarian surface epithelium brushings pooled from ten patients generated on the same array platform [14]. Array data from both cohorts were RMA normalised together using the R package “affy”. Genes that were differentially expressed between ovarian cancer and normal were determined using significance analysis of microarrays [19] where all probes with q-value<5% and fold change >2 were selected as differentially expressed genes. Genes were classified as ‘dysregulated’ in ovarian cancer if they fulfilled the above criteria.

### Comparison of murine ovarian surface epithelium and human ovarian cancer gene expression profiles

Human orthologs of the 905 murine genes found to be differentially expressed in ovarian surface epithelium during the estrous cycle [13] were identified using the list of mouse-human orthologous genes available from the Mouse Genome Informatics database (http://www.informatics.jax.org; accessed Sept 2009). Genes regulated during the estrous cycle which were also dysregulated in ovarian cancer were then identified by matching Entrez Gene IDs and all gene symbols converted to HUGO gene nomenclature symbols. Our final list comprised genes that were regulated during the estrous cycle and shown to be dysregulated in ovarian cancer compared to normal in at least one ovarian cancer dataset.

### Pathway and gene ontology analysis

MetaCore software (St. Joseph, MI, USA) was used to identify the cellular pathways implicated by genes regulated in the estrous cycle and dysregulated in ovarian cancer and to examine whether gene ontologies were statistically over-represented in these gene sets.

### Patient tissue specimens

Details of the patient cohort can be found in Table 1. Cohort 1 consisted of formalin fixed paraffin embedded tissue samples of i) serous ovarian cancer from previously untreated patients (n = 20), ii) normal ovary (n = 10) and matching fallopian tubes (n = 9) from patients who had undergone a prophylactic salpingo-oophorectomy based on a strong family history (n = 6) or who underwent surgery for other non-malignant gynaecological diseases (n = 4), including contralateral benign ovarian tumors in two cases. Cohort 2 comprised 96 cases of serous ovarian cancer with serous ovarian tumor tissue represented on a tissue microarray which included five cases from Cohort 1. The histopathology of representative sections from all cases was reviewed by experienced pathologists (RS and RB) to confirm the diagnosis, histological subtype and to grade the carcinoma cases using standardised criteria [20] as well as to identify tumor areas for construction of the tissue array. Core biopsies (1 mm) of paraffin embedded tumor areas were incorporated into a tissue microarray with 1.5 mm between core centres using a manual arrayer (MTA-II, Beecher Instruments, WI, USA). Each case was represented once on the tissue microarray. A section from the tissue microarray was stained with haematoxylin and eosin to confirm the inclusion of tumor tissue in each core and cores without tumor were excluded from analysis.

**Table 1:** Patient characteristics and clinicopathological features of the cohorts used for immunohistochemical analysis.

	Cohort 1		Cohort 2
	Ovarian cancer	Normal	Ovarian cancer tissue microarray
Number of samples	20	10	96
Median patient age at surgery (range)	60.5 (37–77)	50 (40–60)	57 (22–84)
Histopathological grade^1^			
1	4 (20%)	-	8 (8%)
2	3 (15%)	-	46 (48%)
3	13 (65%)	-	42 (44%)
Stage^2^			
I	5 (25%)	-	3 (3%)
II	1 (5%)	-	5 (5%)
III	10 (50%)	-	78 (81%)
IV	4 (20%)	-	10 (10%)

1 Universal grading system [20].

2 Surgical stage according to International Federation of Gynaecological Oncologists criteria.

#### Clinical Definitions

Surgical staging was assessed in accordance with International Federation of Gynaecological Oncologists classification. Progression-free survival was defined as the time interval between the date of histological diagnosis and the first confirmed sign of disease recurrence or progression based on definitions developed by the Gynaecological Cancer Intergroup as previously described [21]. In the majority of cases the date of progression was assigned using CA125 criteria. In cases where CA125 was not a marker, or progression preceded an increase in CA125, relapse was based on imaging (appearance of new lesion), or, in a minority of cases, global deterioration in health status attributable to the disease. Overall survival was calculated from the date of histological diagnosis to the date of death and censored at last contact date if the patient was alive.

### Immunohistochemistry

Formalin-fixed paraffin embedded sections (3 µm) were mounted on Superfrost Plus microscope slides (Lomb Scientific, NSW, Australia) and dried at 37°C for 1 hr. Sections were dewaxed in histolene and rehydrated through graded ethanols, before being rinsed in water. Slides were then stained with the appropriate antibody using the EnVision+HRP dual link kit (DAKO, Glostrup, Denmark), according to manufacturer's instructions. Briefly, sections were subjected to antigen retrieval using Target Retrieval Solution (DAKO, Glostrup, Denmark) before treatment with 3% H_2_O_2_ for 10 min. Following consecutive rinses with water and PBS, sections were incubated with primary antibody diluted in PBS/0.1% Tween-20 using the dilutions and incubation conditions indicated in Table 2. After rinsing in PBS/0.1% Tween-20 and PBS, sections were incubated for 30 min at room temperature with the Labeled Polymer-HRP solution and then rinsed as previous. Bound antibody was visualised using diamino-benzidine (DAKO, Glostrup, Denmark) prepared according to the manufacturer's instructions. Sections were exposed to diamino-benzidine for 1–2 min and the reaction was stopped in water. Sections were counterstained with Harris' haematoxylin (Amber Scientific, WA, Australia) before dehydration through graded ethanols. Sections were air dried before clearing with histolene and mounting with normount (Fronine, NSW, Australia). To control for non-specific staining, adjacent sections were stained as above, without the primary antibody.

**Table 2:** Details of primary antibodies used.

Antigen	Gene Symbol	Supplier	Catalogue or Clone No.	Dilution	Incubation conditions
Epithelial cell adhesion molecule	EPCAM	Abcam (Cambridge, MA)	clone VU-1D9	1∶100	1 hr at RT^1^
Baculoviral IAP repeat-containing 5	BIRC5	Novus Biologicals (Littleton, CO)	NB500-201	1∶100	1 hr at RT
Mitogen-activated protein kinase 1	MAPK1	Abcam (Cambridge, MA)	clone E460	1∶50	1 hr at RT
Enhancer of zeste homolog 2	EZH2	Zymed (San Francisco, CA)	18-7395	1∶50	1 hr at RT
Lipocalin 2	LCN2	Abcam (Cambridge, MA)	clone HYB 211-01	1∶400	1 hr at RT
SWI/SNF related, matrix associated, actin dependent regulator of chromatin, subfamily a, member 4	SMARCA4	Sigma (St. Louis, MO)	B8184	1∶200	1 hr at RT
p21 protein (Cdc42/Rac)-activated kinase 2	PAK2	Epitomics (Burlingame, CA)	1721-1	1∶50	4°C overnight
NUAK family, SNF1-like kinase, 2	NUAK2	Abgent (San Diego, CA)	AP7158a	1∶100	4°C overnight

1 RT; room temperature.

### Image analysis

Stained sections were analysed using TissueMap (Definiens, Munich, Germany). Briefly, ovarian surface epithelium and inclusion cysts in each section of normal ovary were identified for analysis. A user-defined TissueMap algorithm was used to identify regions of fallopian tube epithelium and tumor tissue based on cell density. Identified areas of ovarian surface epithelium, inclusion cysts, fallopian tube epithelium and tumor tissue were then analysed for the intensity and extent of staining and a histoscore calculated as follows: (% strongly stained cells×3)+(% moderately stained cells×2)+(% weakly stained cells×1)/100, such that scores between 0 and 1 indicated weak staining; 1 and 2 indicated moderate staining; and 2 and 3 indicated strong staining.

### Analyses of copy number aberration and mutation

We compared genes regulated in the estrous cycle and dysregulated in cancer to genes located in regions of copy number aberration (CNA) using data from The Cancer Genome Atlas (TCGA) (http://cancergenome.nih.gov) and a meta-analysis of SNP-based CNA analysis in 398 primary epithelial ovarian cancer samples [22]. Genes within regions of gain (log_2_ copy number ratio >0.3) or loss (log_2_ copy number ratio <−0.3) in greater than 30% of samples in the Broad dataset were downloaded from TCGA data browser (http://tcga-portal.nci.nih.gov/tcga-portal/AnomalySearch.html). Gorringe et al. [22] reported genes within ‘peak’ regions of copy number change as determined by ‘Genomic Identification of Significant Targets in Cancer’ [23] in a subset of 240 samples. ‘Peaks’ represent statistically significant regions of minimal gain or loss, considering both the frequency and amplitude of copy number change, compared to a calculated background aberration rate. Genes within regions of gain (log_2_ copy number ratio >0.3) or loss (log_2_ copy number ratio <−0.3) in greater than 30% of samples were also reported.

We also compared genes regulated in the estrous cycle and dysregulated in cancer to genes commonly mutated in cancer using two previously published datasets [24], [25] as well as the COSMIC database (http://www.sanger.ac.uk/genetics/CGP/cosmic). Futreal et al. [24] compiled a consensus list of genes in which mutations contribute to tumorigenesis, while Greenman et al. [25] screened 518 protein kinase genes and identified an estimated 119 genes with ‘driver’ mutations.

### Statistical analysis

All data were analysed using SPSS (version 16, SPSS, Inc) and a 5% significance level was used throughout. A Chi-square test was used to determine i) if there was a significant overlap in genes differentially expressed in murine ovarian surface epithelium during the estrous cycle and genes differentially expressed in epithelial ovarian cancer compared with normal, and ii) if there was a correlation between gene copy number aberration and direction of differential expression. Paired two-tailed t-tests were used to compare histoscores of ovarian surface epithelium, inclusion cysts and fallopian tube epithelium while a one-way analysis of variance with least squares difference post hoc analysis was used for comparisons with ovarian cancer histoscores. Associations between histoscores and progression-free or overall survival were determined using Kaplan-Meier curves with log-rank test.

## Results

### Ovarian cancer gene expression profiles

We used five publically available ovarian cancer gene expression datasets in our analysis. The selected studies were of either predominantly or exclusively serous subtype, with relatively large numbers of cases, all analysed on an Affymetrix platform and most utilizing ovarian surface epithelium brushings for expression comparison. Details of the published studies are provided in Table 3. The cases analysed were mostly high grade, late stage tumors and where possible we excluded data from borderline and non-serous carcinomas (Table 3). We included results generated from Heinzelmann-Schwarz et al. [17] in our analysis, despite the fact that they compared ovarian cancer tissue to normal whole ovaries, since they integrated their results with 13 other published ovarian cancer expression studies and these results are likely to represent genes consistently highly expressed in ovarian cancer. Importantly, Lu et al. [18] and Heinzelmann-Schwarz et al. [17] only reported genes that were up-regulated in ovarian cancer which introduced a bias into our analyses.

**Table 3:** Published transcription profiling studies used for comparison with mouse ovarian surface epithelium gene profiles.

	Tothill et al. [16]	Bonome et al. [14]	Donninger et al. [15]	Lu et al. [18]	Heinzelmann-Schwarz et al. [17]
No. of specimens	285	80	37	42	51 (+13 other studies)
Histology					
Borderline	18^1^ (6%)	20^1^ (25%)	0	0	8 (16%)
Carcinomas					
Serous	246 (86%)	60 (75%)	37 (100%)	17 (41%)	31 (61%)
Endometrioid	20^1^ (7%)	0	0	9 (21%)	8 (16%)
Clear Cell	0	0	0	7 (17%)	0
Mucinous	0	0	0	9 (21%)	4 (8%)
Adenocarcinoma (NOS)	1^1^ (<0.1%)	0	0	0	0
Grade			not specified		not specified
1	11 (4%)	8 (10%)		3 (7%)	
2	97 (36%)	0		8 (19%)	
3	155 (58%)	72 (90%)		31 (74%)	
unknown	4 (2%)	0		0	
Stage					not specified
I	16 (6%)	14 (18%)	0	16 (38%)	
II	14 (5%)	0	0	5 (12%)	
III	212 (79%)	58 (72%)	Stage III & IV combined	18 (43%)	
IV	21 (8%)	8 (10%)	37 (100%)	3 (7%)	
unknown	4 (2%)	0	0	0	
Tumor content of specimens	≥50%	microdissected tumor tissue	>80%	not specified	>75%
Normal tissue reference^2^	10^3^	10^3^	6	5	4 whole ovaries
Microarray platform (Affy)	U133 Plus 2.0	U133 Plus 2.0	U133 Plus 2.0	U95	GeneChip
No. differentially expressed genes	5868	3479	1084	86	69

1 Borderline and non-serous cases in the Bonome and Tothill datasets were excluded from our analyses.

2 Normal reference sample was ovarian surface epithelial brushings unless otherwise specified.

3 The Tothill et al. [16] & Bonome et al. [14] datasets were compared to an identical normal reference sample.

The total number of genes differentially expressed in ovarian cancer in at least one of the five datasets was 7285. Despite similar cohorts and array platforms there was very little overlap between the datasets - only two genes were overexpressed compared with normal in all five studies (*CD24*, *MAL2*) and only 14 were upregulated in four of the five studies (Table 4). There were 133 genes that were consistently downregulated in the three studies that reported downregulation. The 15 genes with expression that was lowest in ovarian cancer compared with normal are shown in Table 4.

**Table 4:** Genes consistently dysregulated in the ovarian cancer expression datasets examined.

		Fold change/direction of differential expression				
Gene^1^^,^ 2	EOC datasets^3^	Tothill et al. [16]	Bonome et al. [14] 4	Donninger et al. [15]	Lu et al. [18]	Heinzelmann-Schwarz et al. [17]
**Upregulated Genes**						
CD24	5	68.6	47.7	56.2	>3	up
MAL2	5	3.4	3.0	3.1	>3	up
ESRP1	4	8.2	8.1	4.5	-	up
EPCAM	4	7.6	10.4	38.9	-	up
LRIG1	4	7.1	5.9	4.5	>3	-
SPP1	4	7.0	2.6	-	>3	up
WFDC2	4	6.2	17.7	-	>3	up
MTHFD2	4	6.1	6.9	-	>3	up
MUC1	4	5.1	6.1	4.1	>3	-
CP	4	4.7	50.6	18.7	>3	-
PRKCI	4	4.3	2.7	2.2	>3	-
KPNA2	4	4.1	2.4	-	>3	up
VEGFA	4	3.6	2.1	1.6	>3	-
ERBB3	4	3.3	2.6	-	>3	up
KIAA0101	4	2.6	7.3	-	>3	up
SMC4	4	2.3	3.5	2.7	>3	-
**Downregulated Genes**						
ANXA8	3	−23.6	−25.6	−34.4	na	na
CALB2	3	−21.8	−47.6	−57.8	na	na
FAM153C	3	−19.3	−25.0	−27.5	na	na
REEP1	3	−15.7	−25.6	−9.8	na	na
C13orf36	3	−15.4	−20.0	−14.6	na	na
PCOLCE2	3	−11.0	−8.6	−2.7	na	na
LRRN4	3	−10.0	−6.3	−8.2	na	na
EFEMP1	3	−9.2	−16.5	−8.4	na	na
MUM1L1	3	−8.6	−21.7	−5.9	na	na
TCEAL2	3	−8.5	−29.4	−12.8	na	na
MNDA	3	−7.6	−24.4	−8.8	na	na
C8orf84	3	−7.5	−7.7	−8.8	na	na
DPYD	3	−6.7	−15.9	−4.5	na	na
FLRT2	3	−5.5	−30.3	−15.3	na	na
PDGFD	3	−4.8	−14.9	−4.4	na	na

1 Full gene names can be found in Table S1.

2 Genes sorted by number of ovarian cancer datasets showing dysregulation and fold change in Tothill et al. [16].

3 Number of epithelial ovarian cancer (EOC) expression array datasets showing dysregulation. Two ovarian cancer datasets reported upregulated genes only [17], [18].

4 Fold change in late-stage, high grade ovarian cancer relative to normal controls.

### Estrous cycle regulated genes with aberrant expression in ovarian adenocarcinoma

Previously we reported global gene expression changes in pure populations of normal mouse ovarian surface epithelium from immature mice (low hormone levels), cycling mice at proestrus evening (high hormone levels just prior to ovulation), and at estrus morning (low hormone levels just after ovulation) [13]. We found 905 genes regulated, the majority (n = 502; 55%) being regulated on proestrus evening, just prior to ovulation, co-incident with the surge in ovulatory hormones [13]. We compared this list of 905 genes to the 7285 human candidate ovarian cancer genes selected from the five published datasets. Overall, 338 genes that are regulated during the estrous cycle were dysregulated in human ovarian cancer specimens which is a significantly greater overlap than would be expected by chance alone (p<0.0001, Chi square test). Two estrous regulated genes, *EPCAM* and *KIAA0101*, were identified in four of the five published ovarian cancer datasets and 25 genes were identified in three out of five human studies (Table 5), the majority being upregulated in cancer. Almost half of the overlapping genes (11/27, 41%) have a significant number (>5 publications) of prior reports in the literature implicating a role in ovarian cancer, including *NME1*, whereas 13/27, 48% represent novel candidates (Table 5).

**Table 5:** Subset of genes regulated during the murine estrous cycle and dysregulated in ovarian cancer.

Gene^1^	Estrous Stage^2^	EOC datasets^3^	Direction of dysregulation^4^	PubMed hits^5^
EPCAM^6^	PE	4	up	43
KIAA0101	PE/EM	4	up	0
NME1	PE	3	up	53
SPINT2	PE	3	up	24
PTK2	PE	3	up	24
EZR	PE	3	down	13
GATA6	PE	3	down	12
CLDN3	PE	3	up	7
UBE2C	PE	3	up	4
SLC34A2	PE	3	up	3
TPD52	PE	3	up	3
DHCR24	PE	3	up	1
PTPRF	PE/EM	3	up	1
ARF1	PE	3	up	1
CYCS	PE	3	up	0
HSPE1	PE	3	up	0
F11R	PE	3	up	0
HMGB3	PE/EM	3	up	0
ATP11A	PE	3	down	0
NUAK2^6^	PE	3	up	0
CACYBP	PE	3	up	0
PAK1IP1	PE/EM	3	up	0
NAA50	PE	3	up	0
SQLE	PE	3	up	0
CTSC	PE	3	up	0
C5orf34	EM	3	up	0
MUM1L1	PE	3	down	0
EZH2^6^	PE	2	up	4
LCN2^6^	PE	2	up	13
SMARCA4^6^	PE	2	up	6
BIRC5^6^	PE/EM	1	up	109
MAPK1^6^	PE	1	up	78

1 Full gene names can be found in Table S1.

2 Estrous stage specific increase in expression (EM, estrus morning; PE, proestrus evening).

3 Number of epithelial ovarian cancer (EOC) expression array datasets showing dysregulation.

4 Direction of change in ovarian cancer relative to normal controls.

5 Search terms were gene symbol as in Column 1 and “ovarian cancer or ovarian neoplasms [MeSH]” (PubMed accessed Sept 2009).

6 Genes selected for immunohistochemical analysis.

### Immunohistochemical validation of expression in ovarian cancer and normal tissues

Eight candidate genes were selected from the list of 338 genes regulated during the estrous cycle and dysregulated in ovarian cancer for immunohistochemical validation on the basis of known, probable or putative involvement in ovarian cancer as well as availability of antibodies suitable for immunohistochemistry. Two of our chosen genes, *EPCAM* and *BIRC5*, are known to be overexpressed in ovarian cancer and may have therapeutic value [17], [26]–[29] and served as proof-of-principle in our analysis. Five genes, *MAPK1*, *SMARCA4*, *LCN2*, *PAK2* and *EZH2* are known to be involved in other cancer types [30]–[33] but there was little evidence of their involvement in ovarian cancer.

**Figure 1:**
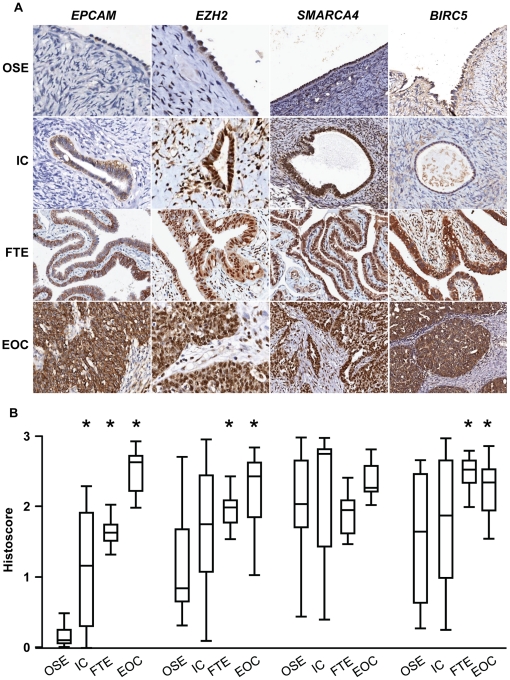
Candidate proteins with high expression in ovarian cancer. **A**. Representative photomicrographs of candidate protein expression in ovarian surface epithelium (OSE), inclusion cyst (IC) and fallopian tube epithelium (FTE) from the same patient and epithelial ovarian cancer (EOC) from a different patient in Cohort 1. **B**. Histoscores of immunostaining results. Significant differences in expression are marked by asterisks (p<0.05). Statistically significant differences are outlined in Table 6.

**Figure 2:**
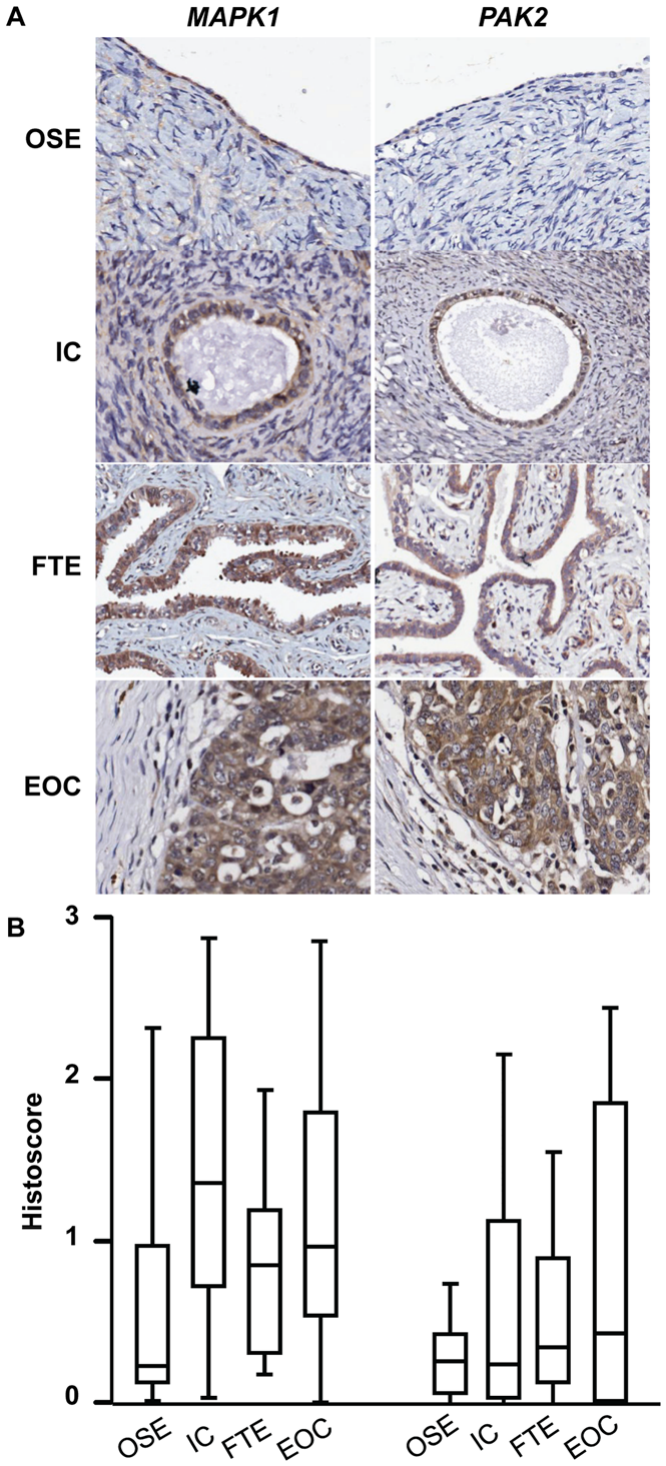
Candidate proteins with low to moderate expression in ovarian cancer. **A**. Representative photomicrographs showing candidate protein expression in ovarian surface epithelium (OSE), inclusion cyst (IC) and fallopian tube epithelium (FTE) from the same patient and epithelial ovarian cancer (EOC) from a different patient in Cohort 1. **B**. Histoscores of immunostaining results. No statistically significant differences were observed.

The protein products of seven of the eight candidate genes were found to be expressed in serous carcinomas. In general, expression of the seven proteins in ovarian surface epithelium and inclusion cysts was quite variable between cases, compared to fallopian tube epithelium and ovarian cancer. EPCAM was expressed at low levels in ovarian surface epithelium and consecutively higher levels in inclusion cysts and fallopian tube epithelium with highest levels seen in ovarian cancer (Figure 1 and Table 6). A similar pattern was observed for EZH2 although expression in ovarian cancer was similar to fallopian tube epithelium (Figure 1 and Table 6). SMARCA4 was expressed at moderate to high levels in all tissues tested (Figure 1) and BIRC5 staining was significantly higher in fallopian tube epithelium and ovarian cancer compared to ovarian surface epithelium and inclusion cysts (Figure 1 and Table 6). Finally, PAK2 and MAPK1 were expressed at low to moderate levels in all tissues tested (Figure 2). Overall, for the majority of proteins, staining was higher in carcinomas compared with ovarian surface epithelium, and in most cases (with the exception of EPCAM), staining in carcinomas was at a similar level to that seen in fallopian tube epithelium. LCN2 staining was not detected at significant levels in the ovarian cancer cohort or in the epithelium of normal tissues, despite expression being increased in ovarian tumors in two expression array analyses [15], [18]. Positive staining was, however, seen inside a few inclusion cysts and in intracytoplasmic vacuoles consistent with LCN2 being a secreted protein (data not shown).

**Table 6:** p-values of significant differences in antigen expression between ovarian surface epithelium, inclusion cysts, fallopian tube epithelium and ovarian cancer^1^.

	Histological Feature				
Antigen^2^		OSE	IC	FTE	EOC
EPCAM	IC	0.0001	-		
	FTE	0.0001	0.03	-	
	EOC	0.0001	0.0001	0.0001	-
EZH2	IC	NS	-		
	FTE	0.011	NS	-	
	EOC	0.0001	0.035	NS	-
BIRC5	IC	NS	-		
	FTE	0.02	0.02	-	-
	EOC	0.02	0.02	NS	-

1 Ovarian surface epithelium (OSE), inclusion cysts (IC) and fallopian tube epithelium (FTE) were from the same patient and were assessed using a paired t-test. Differences in expression between either OSE, IC or FTE from one set of patients and epithelial ovarian cancer (EOC) from a second set of patients were assessed using a one-way ANOVA with least squares difference post-hoc test. NS, not significantly different.

2 Full gene names can be found in Table S1.

### Pathways and gene ontologies

**Figure 3:**
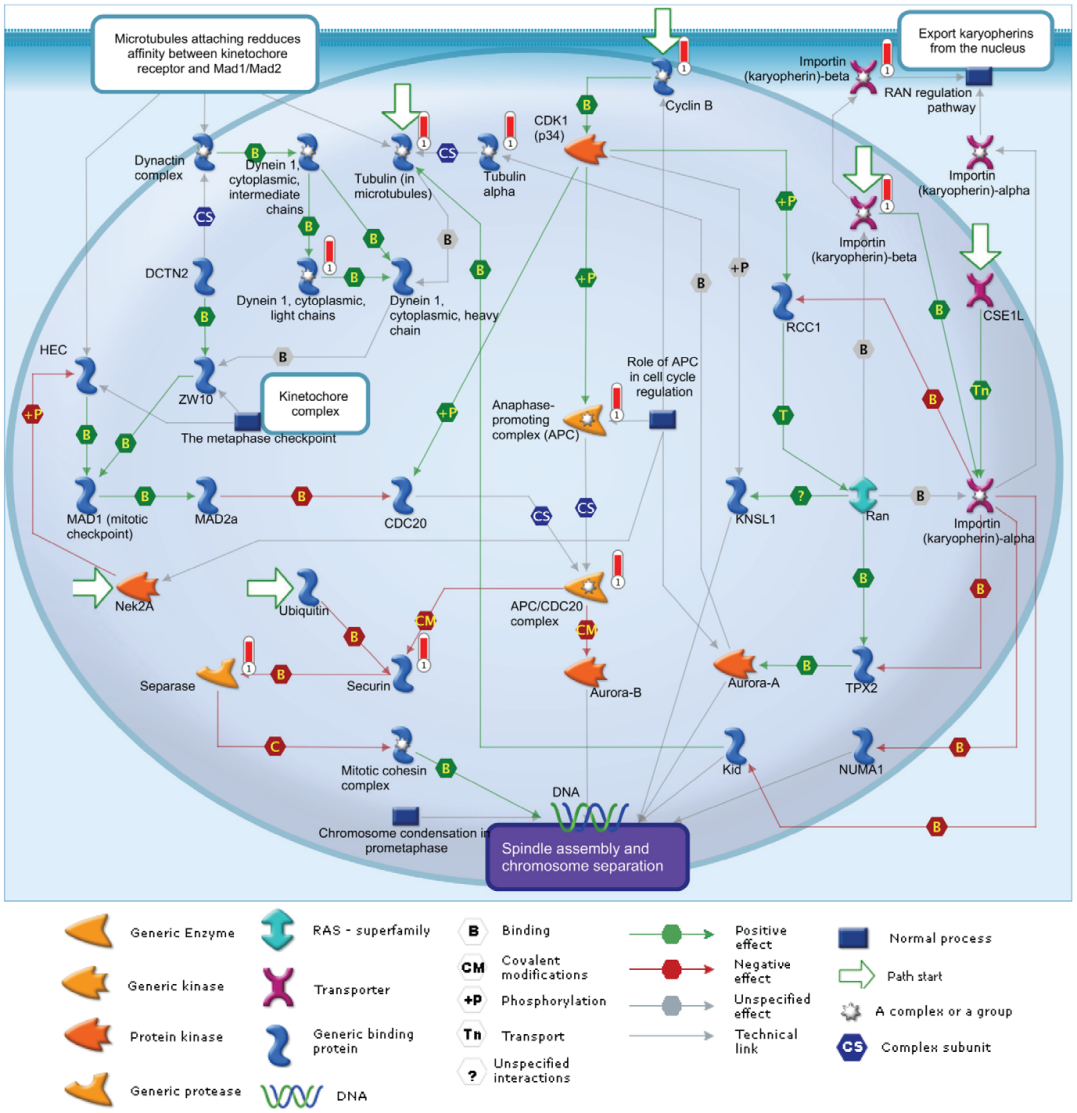
Schematic diagram of most significantly over-represented pathway – spindle assembly and chromosome separation. Genes with vertical red bars adjacent are those which we identified as being regulated during the estrous cycle and are upregulated in ovarian cancer.

We analysed the predicted ontologies of the 338 gene set which overlapped between ovarian cancer specimens and normal ovarian surface epithelium and found over-representation of processes involving protein folding, cytoskeleton, cell cycle and cell adhesion (Table 7). We also analysed known cellular pathways and found the pathways with the highest number of genes from the overlapping set included those involved in cell cycle, endoplasmic reticulum stress and Ras signalling (Table 8). The most significantly over-represented pathway is spindle assembly and chromosome separation (Figure 3).

**Table 7:** Over-represented ontologies^1^ among genes regulated during the estrous cycle and dysregulated in ovarian cancer.

Ontology Network	p-value	Genes^2^
Protein folding in normal condition	2.4×10^−10^	CABIN1; CCT3; CCT7; DNAJB1; DNAJB11; FKBP4; HDAC1; HSP90AA1; HSP90B1; HSPA5/; HSPA9; HSPB1; HSPB8; HSPD1; HSPE1; HSPH1; PFDN2; SERPINH1; ST13; STIP1
Response to unfolded proteins	1.1×10^−9^	DERL1; DNAJB1; HSP90AA1; HSP90B1; HSPA5; HSPA9; HSPB1; HSPB8; HSPD1; HSPE1; HSPH1; SERPINH1; UBE4B; XBP1
Actin filaments	2.8×10^−7^	ACTN1; ACTR2; ARPC1B; CDC42; EZR; FBLIM1; MAPK1; MSN; MYO1C/; MYO1E; PTK2; SPTAN1; TPM4
Spindle microtubules	3.8×10^−6^	BUB1; CCNB1; DYNLL1; EPB41L1; ESPL1; KIF23; KPNB1; PTTG1; TUBA1B; TUBB; TUBGCP2; UBE2C
Regulation of cytoskeleton rearrangement	3.4×10^−5^	ACTN1; ARPC1B; CDC42; EZR; MAPK1; MSN TUBA1B; PTK2; SPTAN1; TUBB
Mitosis	8.2×10^−5^	ANAPC1; BIRC5; BUB1; CCNB1; DYNLL1; ESPL1; F11R; KIF23; KPNB1; NCAPD2; PTTG1; TUBA1B; TUBB
Cell junctions	9.9×10^−5^	ACTN1; FZR1; CLDN3; CLDN7; CTNNA2; KRT8; MAPK1; SPTAN1; TUBA1B; TUBB; WNK4
Integrin-mediated cell-matrix adhesion	2.1×10^−4^	ACTN1; CDC42; DCN; EZR; FBLIM1; JUN; MAPK1; MSN; PTK2; TUBA1B; TUBB
Protein folding in ER and cytoplasm	3.3×10^−4^	EZR/MSN; FKBP4; HSP90AA1; HSPA5; HSPA9; SERPINH1; UGGT1; XBP1
Phagosome in antigen presentation	4.0×10^−4^	ACTN1; C3; CDC42; DERL1; EXOC5; EZR; HSP90AA1; HSP90B1; HSPA5; HSPA9; JUN; MSN; PSMD2

1 Ontology analysis performed using MetaCore software (St. Joseph, MI, USA).

2 Full gene names can be found in Table S1.

**Table 8:** Over-represented pathways^1^ among genes regulated during the estrous cycle and dysregulated in ovarian cancer.

Pathway Category	MetaCore Pathway Maps	p-value	Genes^2^
Cell cycle	Spindle assembly and chromosome separation	1.9×10^−5^	ANAPC1; KPNB1; CCNB1; PTTG1; DYNLL1; TUBA1B; ESPL1; TUBB2C
Apoptosis and survival	Endoplasmic reticulum stress response pathway	3.3×10^−5^	CYCS; JUN; DERL1; PDIA6; HSP90B1; XBP1; HSPA5
G-protein signaling	Ras family GTPases in kinase cascades (scheme)	4.8×10^−5^	CDC42; MAPK1; JUN; NRAS; KRAS
Immune response	Alternative complement pathway	5.1×10^−5^	C3
NA	CFTR folding and maturation (norm and CF)	7.7×10^−5^	DNAJB1; RPN1; HSP90AA1; UGCGL1; HSPA5; HSPA9
Development	Gastrin in cell growth and proliferation	9.2×10^−5^	CDH1; STAT3; JUN; MAPK1; PTK2
Immune response	Lectin induced complement pathway	1.9×10^−4^	C3
Cell cycle	Role of APC in cell cycle regulation	2.1×10^−4^	ANAPC1; CCT7; BUB1; FZR1; CCNB1; CS; CCT3; PTTG1
Immune response	Classical complement pathway	2.6×10^−5^	C3
Development	Leptin signaling via JAK/STAT and MAPK cascades	8.4×10^−5^	CYCS; MAPK1; SOCS3; STAT3

1 Pathway analysis performed using MetaCore software (St. Joseph, MI, USA).

2 Full gene names can be found in Table S1.

### Genes in regions of copy number aberration

Our 338 gene set was further interrogated for genes found in regions of copy number aberration in ovarian cancer using two datasets – The Cancer Genome Atlas and Gorringe et al. [22]. Sixty four of 338 genes (19%) were found in regions of gain or loss in both datasets. The direction of differential expression correlated with copy number aberration for 39/64 genes (61%; p<0.05) (Tables 9 and 10). Most genes which were amplified and upregulated were grouped into 5 genomic regions – 1q, 3q, 8q, 12p and 20q (Table 9). Around half of the deleted and downregulated genes were located on chromosomes 4q and 22q (Table 10). There was a trend for genes within similar chromosomal regions to be co-regulated. For example, 234 patients had copy number gain of at least one of the seven genes in the 20q group. Of these patients, 125 (53%) had amplification of all genes in the 20q group (Table 9).

**Table 9:** Genes regulated during the murine estrous cycle and with putative copy number gain and corresponding upregulation in ovarian cancer.

				Gorringe et al. [22] 4	TCGA^5^	TCGA - Broad data^6^	
Gene^1^	Estrous Stage^2^	EOC datasets^3^	Genomic location	Gain (% cases)	Known CNA	Gain (% cases)	Gain of all genes in group (% cases per group)
*Genes on 1q*							163/243 (67%)
CCT3	PE	2	1q23	36	-	44	
CDC42SE1	PE	1	1q21.1	37	-	47	
S100A6	PE	1	1q21	36	-	45	
*Genes on 3q*							128/355 (36%)
DNAJB11	PE	2	3q27	51	-	64	
PAK2	PE	2	3q29	47	true	58	
SERP1	PE	2	3q25.1	43	-	56	
IGF2BP2	PE/EM	1	3q27.2	52	-	66	
ISY1	PE	1	3q21.3	31	-	41	
RPN1	PE	1	3q21.3	32	true	41	
*Genes on 8q*							148/321 (46%)
SQLE	PE	3	8q24.1	57	-	65	
PTK2	PE	3	8q24.3	55	true	61	
TPD52	PE	3	8q21	32	-	39	
DERL1	PE	2	8q24.13	54	-	63	
*Genes on 12p*							107/236 (45%)
KRAS	PE	2	12p12.1	32	-	40	
FKBP4	PE	2	12p13.33	39	-	36	
NCAPD2	EM	2	12p13.31	37	-	35	
BCAT1	PE/EM	1	12pter-q12	32	-	39	
MGST1	PE	1	12p12.3-p12.1	30	-	36	
*Genes on 20q*							125/234 (53%)
UBE2C	PE	3	20q13.12	37	-	39	
EYA2	PE	2	20q13.1	41	true	41	
AHCY	PE	2	20q11.22	33	-	37	
KIF3B	PE	1	20q11.21	35	true	40	
CTSA	EM	1	20q13.12	37	-	38	
TTPAL	PE	1	20q13.12	34	-	38	
EPB41L1	PE	1	20q11.2-q12	31	-	36	
*Remainder genes in amplified regions*							NA
CLPTM1L	PE	2	5p15.33	31	-	36	
BRD4	EM	1	19	29	true	37	

1 Full gene names can be found in Table S1.

2 Estrus stage specific increase in expression (EM, estrus morning; PE, proestrus evening).

3 Number of epithelial ovarian cancer (EOC) expression array datasets showing dysregulation.

4 Patients (%) with gain (log_2_ CNA >0.3) based on meta-analysis by Gorringe et al. [22] (n = 398).

5 Position of gene within a known region of CNA as reported by TCGA (http://cancergenome.nih.gov).

6 Patients (%) with gain (log_2_ CNA >0.3) based on data from TCGA (n = 568).

**Table 10:** Genes regulated during the murine estrous cycle and with putative copy number loss and corresponding downregulation in ovarian cancer.

				Gorringe et al. [22] 4	TCGA^5^	TCGA - Broad data^6^	
Gene^1^	Estrous Stage^2^	EOC datasets^3^	Genomic location	Loss (% cases)	Known CNA	Loss (% cases)	Loss of all genes in group (% cases per group)
*Genes on 4q*							200/275 (73%)
FAT4	EM	2	4q28.1	35	-	51	
PHF17	PE	2	4q26-q27	34	-	51	
MAPKSP1	PE	1	4q24-q26	34	-	55	
*Genes on 22q*							252/337 (75%)
ST13	PE	1	22q13.2	35	-	70	
TEF	PE/EM	1	22q13.2	34	true	70	
HMOX1	PE	1	22q12	31	-	63	
TIMP3	PE	1	22q12.3	34	-	61	
*Remainder genes in deleted regions*							NA
EZR	PE	3	6q25.3	33	-	51	
CIRBP	PE/EM	2	19p13.3	34	true	77	
EFNB3	EM	1	17p13.1	30	-	66	
IGFBP4	PE/EM	1	17q12-q21.1	30	-	67	
TK2	EM	1	16q22-q23.1	35	-	64	

1 Full gene names can be found in Table S1.

2 Estrus stage specific increase in expression (EM, estrus morning; PE, proestrus evening).

3 Number of epithelial ovarian cancer (EOC) expression array datasets showing dysregulation.

4 Patients (%) with loss (log_2_ CNA <−0.3) based on meta-analysis by Gorringe et al. [22] (n = 398).

5 Position of gene within a known region of CNA as reported by TCGA (http://cancergenome.nih.gov).

6 Patients (%) with loss (log_2_ CNA <−0.3 resp.) based on data from TCGA (n = 568).

### Commonly mutated cancer genes

We interrogated ovulation-related genes which were dysregulated in ovarian cancer for genes commonly mutated in cancer by comparison with data from Futreal et al. [24], Greenman et al. [25] and COSMIC. The analysis in COSMIC was restricted to genes that were i) regulated in the same direction in ovarian cancer in at least three of the previously chosen studies or ii) identified in either Futreal et al. [24], or Greenman et al. [25]. Based on these analyses, we identified 25 genes regulated in mouse ovarian surface epithelium and mutated in cancer including four genes, *SFPQ*, *TPM4*, *MSN* and *SUZ12* which form part of a fusion gene in some cancers (Table 11).

**Table 11:** Genes regulated during the murine estrous cycle, aberrantly expressed in ovarian cancer and putatively mutated in cancer.

				Source of Mutation Data^5^			
					Mutation/Fusion Data from COSMIC^6^		
Gene^1^	Estrous Stage^2^	EOC datasets^3^	Direction of dysregulation^4^	Ref	Ovarian cancer	Other Cancers	Fusion gene partner and site
**Mutated in Ovarian Cancer**							
PTK2	PE	3	up	-	0/26	1/476 CNS, 1/6 skin, 2/226 lung	
NUAK2	PE	3	up	[25]	1/26	1/82 breast	
KRAS	PE	2	up	[24]	377/2754	mutations in multiple organs	
NRAS	PE	2	up	[24]	3/108	mutations in multiple organs	
SMARCA4	PE	2	up	[24]	1/28	mutations in multiple organs	
CDH1	EM	1	up	[24]	1/84	mutations in multiple organs	
BRD4	EM	1	up	[24]	0/26	0/264	
**Mutated in Other Cancers**							
KIAA0101	PE/EM	4	up	-	-	1/22 CNS	
MDM4	PE	3	inconsistent	[24]	-	1/447 CNS, 1/3 aerodigestive tract	
SFPQ	PE/EM	3	inconsistent	[24]	-	1/6 skin	TFE3; kidney and soft tissue
MALAT1	EM	3	down	[24]	no record		
C5orf34	EM	3	up	-	-	1/48 breast	
CYCS	PE	3	up	-	-	1/11 lung	
MUM1L1	PE	3	down	-	-	1/6 skin	
GATA6	PE	3	down	-	-	3/446 CNS	
TPM4	PE	2	up	[24]	-	1/48 breast	ALK; haematopoietic and soft tissue
EZH2	PE	2	up	[24]	-	58/690 haematopoietic tissue, 1/38 intestine, 1/6 skin	
JUN	PE	2	up	[24]	-	0/783	
FOXO1	PE	2	down	[24]	-	1/447 CNS	
DICER1	PE/EM	1	up	[24]	-	1/11 lung, 1/6 skin	
SUZ12	EM	1	up	[24]	-	0/171	JAZF1; endometrial and soft tissue
HSP90AB1	PE	1	up	[24]	-	0/171	
MSN	PE	1	down	[24]	-	0/595	ALK; haematopoietic tissue
RPN1	PE	1	up	[24]	no record		
HNRNPA2B1	PE	1	down	[24]	no record		

1 Full gene names can be found in Table S1.

2 Estrus stage specific increase in expression (EM, estrus morning; PE, proestrus evening).

3 Number of epithelial ovarian cancer (EOC) expression array datasets showing dysregulation.

4 Direction of change in ovarian cancer relative to normal controls.

5 Mutation results based on data from Futreal et al. [24], Greenman et al. [25] and/or Catalogue of Somatic Mutations (COSMIC) database.

6 Organs in which mutations have been found based on data from the Catalogue of Somatic Mutations (COSMIC) database.

**Figure 4:**
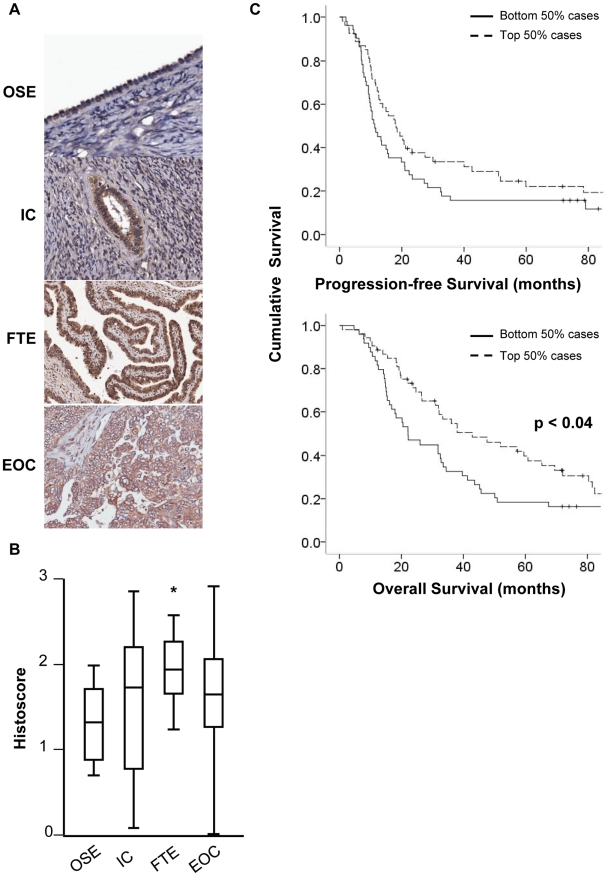
Expression of NUAK2 in malignant ovarian tissue. **A and B**. Representative photomicrographs and histoscores summarising NUAK2 expression in normal ovarian surface epithelium (OSE), inclusion cysts (IC), fallopian tube epithelium (FTE) and epithelial ovarian cancer from both Cohorts 1 and 2. **C**. Kaplan-Meier curves for progression-free and overall survival of ovarian cancer patients dichotomised at median NUAK2 expression. There was no association between NUAK2 expression and progression-free survival (p<0.133), however, lower NUAK2 expression was associated with reduced overall survival (p<0.04) (log-rank test).

*NUAK2* (NUAK family, SNF1-like kinase, 2) was identified in Greenman et al. [25] as a gene with a high probability of having a ‘driver’ mutation in both breast and ovarian carcinomas. We confirmed protein expression of NUAK2 in normal ovarian tissue and analysed the expression of NUAK2 in serous carcinoma, Cohorts 1 and 2, which comprised 20 whole sections and 96 cases on a tissue microarray, respectively. There were five cases common between Cohorts 1 and 2. For these five cases, the histoscore calculated for Cohort 2 was used in all analyses. Amongst this cohort, expression was highly variable (Figure 4). Overall, there were 33 (29%) high, 59 (52%) moderate and 22 (19%) low and there was no association between NUAK2 expression and FIGO stage or histological grade (data not shown). Expression was highest in fallopian tube epithelium although this was only statistically different to NUAK2 staining seen in ovarian surface epithelium (p<0.05; Figure 4). A number of ovarian cancer cases had lost expression relative to fallopian tube epithelium, ovarian surface epithelium and inclusion cysts, although staining in inclusion cysts was highly variable. Although there was no *a priori* evidence to suggest NUAK2 may be associated with outcome, we analysed the expression of NUAK2 for associations with progression-free or overall survival amongst the cohort. We dichotomised the patient cohort at the median histoscore and patients with lower NUAK2 expression had reduced overall survival with median time to death of 22 months compared to patients with higher NUAK2 expression who had median time to death of 42 months (p<0.04) (Figure 4). Patients with lower NUAK2 expression also tended to relapse earlier, however we did not find a significant association between NUAK2 expression and progression-free survival.

## Discussion

During the estrous cycle, ovarian surface epithelium undergo cycles of trauma and proliferation with each ovulation accompanied by hormonal surges and inflammation, which may cause accumulation of genetic damage and ultimately lead to the development of ovarian cancer [1]. Similar hormonal risk factors have been associated with cancer arising in the fallopian tube [34]. In light of this hypothesis, we compared genes with regulated expression in the normal mouse ovarian surface epithelium during the estrous cycle [13], with genes reported to be aberrantly expressed in ovarian cancer in five microarray studies. The five chosen studies combined identified over 7000 genes differentially expressed in ovarian cancer compared to normal ovarian surface epithelium or whole ovaries. Furthermore, as has been previously shown for other microarray datasets [35], there was very little overlap between the five studies despite the similarities in study design. Only *MAL2* and *CD24* were over-expressed in all 5 datasets. *MAL2* is frequently overexpressed in breast carcinoma, and *MAL2* overexpression is associated with gain of the corresponding locus at chromosome 8q24.12. MAL2 binds tumor protein D52 (TPD52), which is over-expressed in ovarian carcinoma, and we have shown that MAL2 is frequently over-expressed in all histological subtypes of ovarian cancer [36]. The fold change in *CD24* was remarkable with ∼50-fold up-regulation observed in three of the five chosen array studies which reported fold change values. Cytoplasmic localisation of CD24 has been shown to be associated with poor survival and CD24 has been investigated as a therapeutic target in ovarian cancer [37],[38]. Recently, CD24 has been investigated in the context of ovarian cancer stem cells although the data are controversial as both the presence and absence of CD24 has been shown to be associated with a stem cell population in ovarian cancer [39], [40].

Here we proposed an inter-study analysis combining results from the five published microarray datasets with our dataset of genes differentially expressed during the murine estrous cycle [13], to address the hypothesis that genes involved in normal ovarian surface epithelium functions, such as ovulation, are aberrantly expressed in ovarian cancer. Identification of this subset may assist in prioritising human candidate genes and pathways implicated in progression to ovarian cancer. We have for the first time, identified genes and pathways that are regulated in ovarian epithelium during the estrous cycle *in vivo* and aberrant in ovarian carcinoma, and have accumulated evidence of involvement in a subset of these genes in ovarian cancer pathogenesis. Overall, 338 genes were found to be regulated during the estrous cycle and dysregulated in human ovarian cancer specimens. Importantly, this overlap was greater than what would be expected by chance alone indicating that the biological processes underpinning the estrous cycle and ovarian cancer are very similar. The vast majority of genes in common were upregulated in the ovarian epithelium of mice during proestrus, just prior to ovulation, when the ovulatory surge results in high levels of cycling hormones. This lends support to a role for ovulatory hormones in ovarian cancer pathogenesis.

Two genes, *EPCAM* and *KIAA0101*, up-regulated on the evening of proestrus, were identified in four of the five human ovarian cancer studies and 25 genes were identified in three of the studies. The expression of genes originally identified in mice was validated in human tissues by immunohistochemistry. We validated the expression of eight genes with varying degrees of evidence for involvement in ovarian cancer. We selected genes with established roles in ovarian cancer (*EPCAM*, *BIRC5*), genes with established roles in cancers other than ovarian cancer (*EZH2*, *SMARCA4*) and genes with limited evidence for involvement in cancer (*MAPK1*, *PAK2*). *EPCAM* and *BIRC5* served as proof of principle in our investigation since they are well known to be overexpressed in ovarian cancer [41], [42] and have been investigated as therapeutic targets [43], [44]. Our results in ovarian cancer agree with previously published reports of high expression of EPCAM localised to the membrane and high expression of BIRC5 evenly distributed between the cytoplasm and nucleus [42]. *EZH2* and *SMARCA4* are likely candidates to be associated with ovarian cancer since they are amplified and/or overexpressed in a number of cancers including prostate, gastric, and breast [33], [45], [46]. This study is the first to demonstrate EZH2 and SMARCA4 expression at a protein level in ovarian tissues. There is limited evidence for the involvement of MAPK1 in cancer beyond *in vitro* studies while PAK2 has been shown to be expressed in ovarian cancer in one report and interacts with known cancer-associated genes [47], [48]. Our data confirm that MAPK1 and PAK2 are expressed in normal and malignant ovarian tissue.

The general pattern of low expression in ovarian surface epithelium and higher expression in inclusion cysts seen in our study has been previously reported in other studies [9]. It is thought that high hormone levels in the ovarian stroma may induce expression of a range of genes in the epithelium lining inclusion cysts. We also observed a large variability in the expression of our candidate proteins in normal ovarian surface epithelium and inclusion cysts compared to that seen in fallopian tube epithelium and ovarian cancer. Given that these genes were originally identified as differentially expressed during the murine estrous cycle, we would hypothesize that these genes are hormonally regulated as has already been shown for *BIRC5* [49], [50]. The variability of expression seen in normal ovarian surface epithelium and inclusion cysts may reflect the varying hormonal status of the women in Cohort 1 at the time of tissue collection. It is likely that some of the women in Cohort 1 are pre-menopausal given that half the women are under 50 years of age, however, the exact menopausal status of the women in Cohort 1 is unknown.

An unexpected finding of this study is the relatively similar expression of most of our candidate genes in fallopian tube epithelium and ovarian cancer. It is possible that similar expression of these genes is a reflection of the phenotypic similarity between serous ovarian cancer and fallopian tube epithelium, therefore, their contribution to ovarian tumorigenesis cannot be discerned from our data. While expression levels in fallopian tube epithelium and ovarian cancer were similar for most of our candidate genes, it is important to note that those genes which harbour mutations may exert a tumorigenic effect without an appreciable change in expression levels. This is particularly relevant for *SMARCA4* which is mutated in many cancer cell lines as well as patient samples [51] but was not significantly overexpressed at a protein level in ovarian cancer compared to normal tissues. LCN2 was not detected in our cohort of serous ovarian cancer which is likely due to the predominance of high grade cases in our cohort since Lim et al. [52] have shown that LCN2 was expressed at low levels in high grade ovarian cancer but moderate to strong levels in tumors of low grade and/or low malignant potential.

Gene ontology and pathway analysis found statistically significant over-representation of a number of processes and pathways associated with tumorigenesis, including apoptosis, cell adhesion and cell cycle. In total, 13 of the 27 genes identified in three or more array datasets, have previously been described by independent studies to be differentially expressed in ovarian cancer specimens, including *EPCAM*, *CLDN3*, *PTK2* and *TPD52* while a further 11 genes have been investigated in the context of other cancers. Interestingly, *KIAA0101*, *CANX* and *NME1* have all been found to be highly expressed in at least eight different carcinomas, including breast, lung and prostate [53], representing possible global tumor markers. However further work is required to validate these results. The remaining three genes *NAT13*, *MUM1L1*, *C5orf34*, however, have yet to be investigated in any cancer to our knowledge thus validating our approach for the purposes of identifying novel ovarian cancer-associated genes.

Of the genes found to be regulated during the estrous cycle and mutated in cancers, 15 have been described in ovarian cancer including *KRAS* which has an established role in ovarian cancer, particularly in Type I tumors, that is low-grade ovarian cancer and low malignant potential tumors [54]. In addition, *FOXO1* is a cisplatin sensitivity gene in cell lines [55] and *MSN*, was identified as a novel diagnostic marker for distinguishing ovarian cancer from colon cancer [56]. The remaining 10 genes have not been investigated in ovarian cancer and include *SFPQ* which has been suggested to function as an androgen receptor co-regulator [57]. These data provide further example of the potential utility of our approach of using the ovarian surface epithelium gene signatures to characterise genes and pathways implicated in ovarian cancer.

Based on our microarray and *in silico* analyses, *NUAK2* is regulated during the murine estrous cycle, dysregulated in ovarian cancer and putatively contains a driver mutation in ovarian and breast cancer. Although it has not been specifically investigated in the context of cancer, there is evidence that *NUAK2* may be involved in cancer-associated pathways and may have pro-survival activity [58]. We extended our immunohistochemical validation to an ovarian cancer tissue microarray with ∼100 specimens. This is the first study to show NUAK2 expression in ovarian tissue. Interestingly, a proportion of malignant cases expressed NUAK2 at reduced levels compared to normal fallopian tube epithelium and inclusion cysts. Although our primary aim was to identify genes, which may be involved in development of ovarian cancer, aberrations in pathways which confer a survival advantage and promote tumor development may also contribute to survival in response to therapy and therefore may also be associated with outcome. Indeed, expression of NUAK2 was significantly associated with overall survival with median time to death differing by 20 months between median dichotomised groups of patients. Patients with low NUAK2 expression fared worse than patients with high expression of NUAK2. The putative driver mutation in *NUAK2* and association of loss of expression with reduced overall survival suggests NUAK2 may have tumour suppressive activity. Our data suggest that *NUAK2* warrants further investigation in *in vitro* functional models of ovarian cancer pathogenesis.

Our *in silico* analyses have identified a number of candidates including genes with evidence of both copy number aberration and mutation. Amongst these is *KRAS* which, as aforementioned, has an established role in ovarian cancer [54]. Similarly, PTK2 is also amplified in ovarian cancer and mutated in solid tumors. Overexpression of PTK2 in ovarian cancer is significantly associated with poorer survival [59] and PTK2 is being investigated as a therapeutic target in xenograft models of ovarian cancer [60]. Amongst the genes with mutations in solid tumors is *BUB1*, for which there is no existing literature in the context of ovarian cancer. *BUB1* is a component of the spindle assembly checkpoint pathway which is critical for ensuring correct chromosome segregation and prevention of aneuploidy. The genes we identified which are regulated during the estrous cycle and dysregulated in cancer are over-represented in two pathways associated with spindle assembly. Defects in spindle assembly checkpoint proteins, including BUB1, are sufficient to allow proliferation of BRCA2 deficient cells which in the absence of a “second-hit” do not have a growth advantage [61]. While little work has been done on BUB1 itself, other members of the spindle assembly checkpoint have been investigated in the context of ovarian cancer including BUBR1, which is an independent prognostic indicator for ovarian cancer [62]. Furthermore, a functioning spindle assembly checkpoint is required for sensitivity to microtubule inhibiting drugs including paclitaxel which is widely used in ovarian cancer [63]. *NCAPD2*, a candidate gene we identified with copy number aberration and mutation, is a component of the condensin complex which is involved in resolution and segregation of sister chromatids during mitosis [64]. It is interesting that both *BUB1* and *NCAPD2* have emerged as candidate genes in our analyses which perhaps indicates the importance of aberrations in the chromosomal segregation pathway for ovarian cancer development.

Amongst genes we identified with copy number gain is *ARPC1B* which is expressed in spontaneously transformed tumorigenic mouse ovarian surface epithelial cell lines and is positively correlated with tumor load in a mouse model of ovarian cancer [65]. *Ezrin*, has been investigated in a large study of ovarian cancer where its expression was reduced in 440 ovarian cancer samples compared to normal and lower expression was associated with higher grade and shorter survival although not in a multivariate analysis [66]. Eyes absent 2 (*EYA2*) is upregulated in ovarian cancer compared to normal ovarian surface epithelium in part due to genomic amplification. *EYA2* functions as a transcriptional coactivator in ovarian cancer cell lines and ectopic expression of *EYA2* promotes growth of ovarian cancer xenografts. High expression of *EYA2* is significantly associated with a shorter overall survival in late stage cancers [67]. The identification of genes with at least putative roles in ovarian cancer validates our approach of a multi-*in silico* analysis approach for prioritising candidate genes for ovarian tumorigenesis. Another novel candidate with copy number loss, *CIRBP*, is a cold-inducible protein, however, it is also induced by UV irradiation and hypoxia [68], [69]. Evidence for a role for *CIRBP* in cancer is complex with some studies indicating overexpression confers a growth advantage [70] while others report downregulation or complete loss in tumor tissue samples [71]. Interestingly while overexpression confers a growth advantage, loss enhances sensitivity to DNA damaging agents. In our analyses, *CIRBP* is downregulated in proestrus evening and restored in estrus morning, downregulated in expression array studies and lost in array CNA studies of ovarian cancer. Given the limited evidence it is difficult to hypothesise a role for *CIRBP* in ovarian cancer. However, it is tempting to speculate that loss of *CIRBP* in ovarian surface epithelium may result in increased susceptibility to the DNA damaging effects of hormones thereby increasing risk of tumor initiation.

Using a data mining approach we have identified that genes involved in the normal processes of the ovarian cycle may constitute potentially important signalling pathways involved in ovarian cancer. Taken together, these results further support the existing evidence that genes involved in normal cellular pathways during the ovulatory cycle, are also potential candidates in epithelial ovarian carcinogenesis and worthy of additional research.

## Supporting Information

Table S1 **Full names of genes identified in the manuscript.**
(DOC)

## References

[1] Fathalla MF (1971). Incessant ovulation-a factor in ovarian
neoplasia?. Lancet.

[2] Risch HA (1998). Hormonal etiology of epithelial ovarian cancer, with a hypothesis
concerning the role of androgens and progesterone.. J Natl Cancer Inst.

[3] Banks E, Beral V, Reeves G (1997). The epidemiology of epithelial ovarian cancer: a
review.. Int J Gynecol Cancer.

[4] Casagrande JT, Louie EW, Pike MC, Roy S, Ross RK (1979). “Incessant ovulation” and ovarian
cancer.. Lancet.

[5] Zheng W, Lu JJ, Luo F, Zheng Y, Feng Y (2000). Ovarian epithelial tumor growth promotion by follicle-stimulating
hormone and inhibition of the effect by luteinizing hormone.. Gynecol Oncol.

[6] Choi JH, Wong AS, Huang HF, Leung PC (2007). Gonadotropins and ovarian cancer.. Endocr Rev.

[7] Jernstrom H, Borg K, Olsson H (2005). High follicular phase luteinizing hormone levels in young healthy
BRCA1 mutation carriers: implications for breast and ovarian cancer
risk.. Mol Genet Metab.

[8] Rodriguez GC, Walmer DK, Cline M, Krigman H, Lessey BA (1998). Effect of progestin on the ovarian epithelium of macaques: cancer
prevention through apoptosis?. J Soc Gynecol Investig.

[9] Blaustein A, Kaganowicz A, Wells J (1982). Tumor markers in inclusion cysts of the ovary.. Cancer.

[10] Lee Y, Miron A, Drapkin R, Nucci MR, Medeiros F (2007). A candidate precursor to serous carcinoma that originates in the
distal fallopian tube.. J Pathol.

[11] Tone AA, Begley H, Sharma M, Murphy J, Rosen B (2008). Gene expression profiles of luteal phase fallopian tube
epithelium from BRCA mutation carriers resemble high-grade serous
carcinoma.. Clin Cancer Res.

[12] Armaiz-Pena GN, Mangala LS, Spannuth WA, Lin YG, Jennings NB (2009). Estrous cycle modulates ovarian carcinoma growth.. Clin Cancer Res.

[13] Gava N, Clarke CL, Bye C, Byth K, deFazio A (2008). Global gene expression profiles of ovarian surface epithelial
cells in vivo.. J Mol Endocrinol.

[14] Bonome T, Lee J-Y, Park D-C, Radonovich M, Pise-Masison C (2005). Expression profiling of serous low malignant potential,
low-grade, and high-grade tumors of the ovary.. Cancer Res.

[15] Donninger H, Bonome T, Radonovich M, Pise-Masison CA, Brady J (2004). Whole genome expression profiling of advance stage papillary
serous ovarian cancer reveals activated pathways.. Oncogene.

[16] Tothill RW, Tinker AV, George J, Brown R, Fox SB (2008). Novel molecular subtypes of serous and endometrioid ovarian
cancer linked to clinical outcome.. Clin Cancer Res.

[17] Heinzelmann-Schwarz VA, Gardiner-Garden M, Henshall SM, Scurry J, Scolyer RA (2004). Overexpression of the cell adhesion molecules DDR1, Claudin 3,
and Ep-CAM in metaplastic ovarian epithelium and ovarian
cancer.. Clin Cancer Res.

[18] Lu KH, Patterson AP, Wang L, Marquez RT, Atkinson EN (2004). Selection of potential markers for epithelial ovarian cancer with
gene expression arrays and recursive descent partition
analysis.. Clin Cancer Res.

[19] Tusher VG, Tibshirani R, Chu G (2001). Significance analysis of microarrays applied to the ionizing
radiation response.. PNAS.

[20] Shimizu Y, Kamoi S, Amada S, Akiyama F, Silverberg SG (1998). Toward the development of a universal grading system for ovarian
epithelial carcinoma: testing of a proposed system in a series of 461
patients with uniform treatment and follow-up.. Cancer.

[21] Vergote I, Rustin GJ, Eisenhauer EA, Kristensen GB, Pujade-Lauraine E (2000). Re: new guidelines to evaluate the response to treatment in solid
tumors [ovarian cancer]. Gynecologic Cancer
Intergroup.. J Natl Cancer Inst.

[22] Gorringe KL, George J, Anglesio MS, Ramakrishna M, Etemadmoghadam D (2010). Copy number analysis identifies novel interactions between
genomic loci in ovarian cancer.. PLoS ONE.

[23] Beroukhim R, Getz G, Nghiemphu L, Barretina J, Hsueh T (2007). Assessing the significance of chromosomal aberrations in cancer:
methodology and application to glioma.. PNAS.

[24] Futreal PA, Coin L, Marshall M, Down T, Hubbard T (2004). A census of human cancer genes.. Nat Rev Cancer.

[25] Greenman C, Stephens P, Smith R, Dalgliesh GL, Hunter C (2007). Patterns of somatic mutation in human cancer
genomes.. Nature.

[26] Ferrandina G, Legge F, Martinelli E, Ranelletti FO, Zannoni GF (2005). Survivin expression in ovarian cancer and its correlation with
clinico-pathological, surgical and apoptosis-related
parameters.. Br J Cancer.

[27] Spizzo G, Went P, Dirnhofer S, Obrist P, Moch H (2006). Overexpression of epithelial cell adhesion molecule (Ep-CAM) is
an independent prognostic marker for reduced survival of patients with
epithelial ovarian cancer.. Gynecol Oncol.

[28] Sui L, Dong Y, Ohno M, Watanabe Y, Sugimoto K (2002). Survivin expression and its correlation with cell proliferation
and prognosis in epithelial ovarian tumors.. Int J Oncol.

[29] Takai N, Miyazaki T, Nishida M, Nasu K, Miyakawa I (2002). Expression of survivin is associated with malignant potential in
epithelial ovarian carcinoma.. Int J Mol Med.

[30] Hoshino R, Chatani Y, Yamori T, Tsuruo T, Oka H (1999). Constitutive activation of the 41-/43-kDa mitogen-activated
protein kinase signaling pathway in human tumors.. Oncogene.

[31] Reisman DN, Sciarrotta J, Wang W, Funkhouser WK, Weissman BE (2003). Loss of BRG1/BRM in human lung cancer cell lines and primary lung
cancers: correlation with poor prognosis.. Cancer Res.

[32] Stoesz SP, Friedl A, Haag JD, Lindstrom MJ, Clark GM (1998). Heterogeneous expression of the lipocalin NGAL in primary breast
cancers.. Int J Cancer.

[33] Varambally S, Dhanasekaran SM, Zhou M, Barrette TR, Kumar-Sinha C (2002). The polycomb group protein EZH2 is involved in progression of
prostate cancer.. Nature.

[34] Jordan SJ, Green AC, Whiteman DC, Moore SP, Bain CJ (2008). Serous ovarian, fallopian tube and primary peritoneal cancers: a
comparative epidemiological analysis.. Int J Cancer.

[35] Gyorffy B, Schafer R (2008). Meta-analysis of gene expression profiles related to relapse-free
survival in 1,079 breast cancer patients.. Breast Cancer Res Treat.

[36] Byrne JA, Maleki S, Hardy JR, Gloss BS, Murali R (2010). MAL2 and tumor protein D52 (TPD52) are frequently overexpressed
in ovarian carcinoma, but differentially associated with histological
subtype and patient outcome.. BMC Cancer.

[37] Kristiansen G, Denkert C, Schluns K, Dahl E, Pilarsky C (2002). CD24 is expressed in ovarian cancer and is a new independent
prognostic marker of patient survival.. Am J Pathol.

[38] Su D, Deng H, Zhao X, Zhang X, Chen L (2009). Targeting CD24 for treatment of ovarian cancer by short hairpin
RNA.. Cytotherapy.

[39] Gao MQ, Choi YP, Kang S, Youn JH, Cho NH (2010). CD24+ cells from hierarchically organized ovarian cancer are
enriched in cancer stem cells.. Oncogene.

[40] Shi MF, Jiao J, Lu WG, Ye F, Ma D (2010). Identification of cancer stem cell-like cells from human
epithelial ovarian carcinoma cell line.. Cell Mol Life Sci.

[41] Drapkin R, Crum CP, Hecht JL (2004). Expression of candidate tumor markers in ovarian carcinoma and
benign ovary: Evidence for a link between epithelial phenotype and
neoplasia.. Hum Pathol.

[42] Tringler B, Lehner R, Shroyer AL, Shroyer KR (2004). Immunohistochemical localization of survivin in serous tumors of
the ovary.. Appl Immunohistochem Mol Morphol.

[43] Burges A, Wimberger P, Kumper C, Gorbounova V, Sommer H (2007). Effective relief of malignant ascites in patients with advanced
ovarian cancer by a trifunctional anti-EpCAM x anti-CD3 antibody: a phase
I/II study.. Clin Cancer Res.

[44] Caldas H, Jaynes FO, Boyer MW, Hammond S, Altura RA (2006). Survivin and Granzyme B-induced apoptosis, a novel anticancer
therapy.. Mol Cancer Ther.

[45] Kleer CG, Cao Q, Varambally S, Shen R, Ota I (2003). EZH2 is a marker of aggressive breast cancer and promotes
neoplastic transformation of breast epithelial cells.. PNAS.

[46] Sentani K, Oue N, Kondo H, Kuraoka K, Motoshita J (2001). Increased expression but not genetic alteration of BRG1, a
component of the SWI/SNF complex, is associated with the advanced stage of
human gastric carcinomas.. Pathobiology.

[47] Siu MK, Wong ES, Chan HY, Kong DS, Woo NW (2009). Differential expression and phosphorylation of Pak1 and Pak2 in
ovarian cancer: Effects on prognosis and cell invasion.. Int J Cancer.

[48] Steinmetz R, Wagoner HA, Zeng P, Hammond JR, Hannon TS (2004). Mechanisms regulating the constitutive activation of the
extracellular signal-regulated kinase (ERK) signaling pathway in ovarian
cancer and the effect of ribonucleic acid interference for ERK1/2 on cancer
cell proliferation.. Mol Endocrinol.

[49] Huang Y, Jin H, Liu Y, Zhou J, Ding J (2010). Follicle-stimulating hormone inhibits ovarian cancer cell
apoptosis by upregulating survivin and downregulating PDCD6 and
DR5.. Endocr Relat Cancer.

[50] Nabilsi NH, Broaddus RR, McCampbell AS, Lu KH, Lynch HT (2010). Sex hormone regulation of survivin gene
expression.. J Endocrinol.

[51] Wong AK, Shanahan F, Chen Y, Lian L, Ha P (2000). BRG1, a component of the SWI-SNF complex, is mutated in multiple
human tumor cell lines.. Cancer Res.

[52] Lim R, Ahmed N, Borregaard N, Riley C, Wafai R (2007). Neutrophil gelatinase-associated lipocalin (NGAL) an
early-screening biomarker for ovarian cancer: NGAL is associated with
epidermal growth factor-induced epithelio-mesenchymal
transition.. Int J Cancer.

[53] Pilarsky C, Wenzig M, Specht T, Saeger HD, Grutzmann R (2004). Identification and validation of commonly overexpressed genes in
solid tumors by comparison of microarray data.. Neoplasia.

[54] Shih Ie M, Kurman RJ (2004). Ovarian tumorigenesis: a proposed model based on morphological
and molecular genetic analysis.. Am J Pathol.

[55] Arimoto-Ishida E, Ohmichi M, Mabuchi S, Takahashi T, Ohshima C (2004). Inhibition of phosphorylation of a forkhead transcription factor
sensitizes human ovarian cancer cells to cisplatin.. Endocrinology.

[56] Nishizuka S, Chen S-T, Gwadry FG, Alexander J, Major SM (2003). Diagnostic markers that distinguish colon and ovarian
adenocarcinomas: Identification by genomic, proteomic, and tissue array
profiling.. Cancer Res.

[57] Kuwahara S, Ikei A, Taguchi Y, Tabuchi Y, Fujimoto N (2006). PSPC1, NONO, and SFPQ are expressed in mouse Sertoli cells and
may function as coregulators of androgen receptor-mediated
transcription.. Biol Reprod.

[58] Legembre P, Schickel R, Barnhart BC, Peter ME (2004). Identification of SNF1/AMP kinase-related kinase as an
NF-kappaB-regulated anti-apoptotic kinase involved in CD95-induced motility
and invasiveness.. J Biol Chem.

[59] Sood AK, Coffin JE, Schneider GB, Fletcher MS, DeYoung BR (2004). Biological significance of focal adhesion kinase in ovarian
cancer: role in migration and invasion.. Am J Pathol.

[60] Shahzad MM, Lu C, Lee JW, Stone RL, Mitra R (2009). Dual targeting of EphA2 and FAK in ovarian
carcinoma.. Cancer Biol Ther.

[61] Lee H, Trainer AH, Friedman LS, Thistlethwaite FC, Evans MJ (1999). Mitotic checkpoint inactivation fosters transformation in cells
lacking the breast cancer susceptibility gene, Brca2.. Mol Cell.

[62] Lee YK, Choi E, Kim MA, Park PG, Park NH (2009). BubR1 as a prognostic marker for recurrence-free survival rates
in epithelial ovarian cancers.. Br J Cancer.

[63] Lee EA, Keutmann MK, Dowling ML, Harris E, Chan G (2004). Inactivation of the mitotic checkpoint as a determinant of the
efficacy of microtubule-targeted drugs in killing human cancer
cells.. Mol Cancer Ther.

[64] Watrin E, Legagneux V (2005). Contribution of hCAP-D2, a non-SMC subunit of condensin I, to
chromosome and chromosomal protein dynamics during mitosis.. Mol Cell Biol.

[65] Urzua U, Roby KF, Gangi LM, Cherry JM, Powell JI (2006). Transcriptomic analysis of an in vitro murine model of ovarian
carcinoma: functional similarity to the human disease and identification of
prospective tumoral markers and targets.. J Cell Physiol.

[66] Moilanen J, Lassus H, Leminen A, Vaheri A, Butzow R (2003). Ezrin immunoreactivity in relation to survival in serous ovarian
carcinoma patients.. Gynecol Oncol.

[67] Zhang L, Yang N, Huang J, Buckanovich RJ, Liang S (2005). Transcriptional coactivator Drosophila eyes absent homologue 2 is
up-regulated in epithelial ovarian cancer and promotes tumor
growth.. Cancer Res.

[68] Sheikh MS, Carrier F, Papathanasiou MA, Hollander MC, Zhan Q (1997). Identification of several human homologs of hamster DNA
damage-inducible transcripts. Cloning and characterization of a novel
UV-inducible cDNA that codes for a putative RNA-binding
protein.. J Biol Chem.

[69] Wellmann S, Buhrer C, Moderegger E, Zelmer A, Kirschner R (2004). Oxygen-regulated expression of the RNA-binding proteins RBM3 and
CIRP by a HIF-1-independent mechanism.. J Cell Sci.

[70] Artero-Castro A, Callejas FB, Castellvi J, Kondoh H, Carnero A (2009). Cold-inducible RNA-binding protein bypasses replicative
senescence in primary cells through extracellular signal-regulated kinase 1
and 2 activation.. Mol Cell Biol.

[71] Hamid AA, Mandai M, Fujita J, Nanbu K, Kariya M (2003). Expression of cold-inducible RNA-binding protein in the normal
endometrium, endometrial hyperplasia, and endometrial
carcinoma.. Int J Gynecol Pathol.

