# Supplementary material for: Comparison of Expression Profiles in Ovarian Epithelium In Vivo and Ovarian Cancer Identifies Novel Candidate Genes Involved in Disease Pathogenesis
Source: PLoS One. 2011 Mar 15;6(3):e17617. doi: 10.1371/journal.pone.0017617 (PMC3057977; doi:10.1371/journal.pone.0017617)
Supplement: Table S1 — Full names of genes identified in the manuscript. (DOC) [file pone.0017617.s001.doc]

| **Gene Symbol** | **Gene Name** |
| --- | --- |
| ACTN1 | actinin, alpha 1 |
| ACTR2 | ARP2 actin-related protein 2 homolog (yeast) |
| AHCY | adenosylhomocysteinase |
| ANAPC1 | anaphase promoting complex subunit 1 |
| ANXA8 | annexin A8 |
| ARF1 | ADP-ribosylation factor 1 |
| ARPC1B | actin related protein 2/3 complex, subunit 1B, 41kDa |
| ATP11A | ATPase, class VI, type 11A |
| BCAT1 | branched chain amino-acid transaminase 1, cytosolic |
| BIRC5 | baculoviral IAP repeat-containing 5 |
| BRD4 | bromodomain containing 4 |
| BUB1 | budding uninhibited by benzimidazoles 1 homolog (yeast) |
| C3 | complement component 3 |
| C5orf34 | chromosome 5 open reading frame 34 |
| C8orf84 | chromosome 8 open reading frame 84 |
| C13orf36 | chromosome 13 open reading frame 36 |
| CABIN1 | calcineurin binding protein 1 |
| CACYBP | calcyclin binding protein |
| CALB2 | calbindin 2 |
| CCNB1 | cyclin B1 |
| CCT3 | chaperonin containing TCP1, subunit 3 (gamma) |
| CCT7 | chaperonin containing TCP1, subunit 7 (eta) |
| CD24 | CD24 molecule |
| CDC42 | cell division cycle 42 (GTP binding protein, 25kDa) |
| CDC42SE1 | CDC42 small effector 1 |
| CDH1 | cadherin 1, type 1, E-cadherin (epithelial) |
| CIRBP | cold inducible RNA binding protein |
| CLDN3 | claudin 3 |
| CLDN7 | claudin 7 |
| CLPTM1L | CLPTM1-like |
| CP | ceruloplasmin (ferroxidase) |
| CS | citrate synthase |
| CTNNA2 | catenin (cadherin-associated protein), alpha 2 |
| CTSA | cathepsin A |
| CTSC | cathepsin C |
| CYCS | cytochrome c, somatic |
| DCN | decorin |
| DERL1 | Der1-like domain family, member 1 |
| DHCR24 | 24-dehydrocholesterol reductase |
| DICER1 | dicer 1, ribonuclease type III |
| DNAJB1 | DnaJ (Hsp40) homolog, subfamily B, member 1 |
| DNAJB11 | DnaJ (Hsp40) homolog, subfamily B, member 11 |
| DPYD | dihydropyrimidine dehydrogenase |
| DYNLL1 | dynein, light chain, LC8-type 1 |
| EFEMP1 | EGF-containing fibulin-like extracellular matrix protein 1 |
| EFNB3 | ephrin-B3 |
| EPB41L1 | erythrocyte membrane protein band 4.1-like 1 |
| EPCAM | epithelial cell adhesion molecule |
| ERBB3 | v-erb-b2 erythroblastic leukemia viral oncogene homolog 3 (avian) |
| ESPL1 | extra spindle pole bodies homolog 1 (S. cerevisiae) |
| ESRP1 | epithelial splicing regulatory protein 1 |
| EXOC5 | exocyst complex component 5 |
| EYA2 | eyes absent homolog 2 (Drosophila) |
| EZH2 | enhancer of zeste homolog 2 (Drosophila) |
| EZR | ezrin |
| F11R | F11 receptor |
| FAM153C | family with sequence similarity 153, member C |
| FAT4 | FAT tumor suppressor homolog 4 (Drosophila) |
| FBLIM1 | filamin binding LIM protein 1 |
| FKBP4 | FK506 binding protein 4, 59kDa |
| FLRT2 | fibronectin leucine rich transmembrane protein 2 |
| FOXO1 | forkhead box O1 |
| FZR1 | fizzy/cell division cycle 20 related 1 (Drosophila) |
| GATA6 | GATA binding protein 6 |
| HDAC1 | histone deacetylase 1 |
| HMGB3 | high-mobility group box 3 |
| HMOX1 | heme oxygenase (decycling) 1 |
| HNRNPA2B1 | heterogeneous nuclear ribonucleoprotein A2/B1 |
| HSP90AA1 | heat shock protein 90kDa alpha (cytosolic), class A member 1 |
| HSP90AB1 | heat shock protein 90kDa alpha (cytosolic), class B member 1 |
| HSP90B1 | heat shock protein 90kDa beta (Grp94), member 1 |
| HSPA5 | heat shock 70kDa protein 5 (glucose-regulated protein, 78kDa) |
| HSPA9 | heat shock 70kDa protein 9 (mortalin) |
| HSPB1 | heat shock 27kDa protein 1 |
| HSPB8 | heat shock 22kDa protein 8 |
| HSPD1 | heat shock 60kDa protein 1 (chaperonin) |
| HSPE1 | heat shock 10kDa protein 1 (chaperonin 10) |
| HSPH1 | heat shock 105kDa/110kDa protein 1 |
| IGF2BP2 | insulin-like growth factor 2 mRNA binding protein 2 |
| IGFBP4 | insulin-like growth factor binding protein 4 |
| ISY1 | ISY1 splicing factor homolog (S. cerevisiae) |
| JUN | jun oncogene |
| KIAA0101 | KIAA0101 |
| KIF23 | kinesin family member 23 |
| KIF3B | kinesin family member 3B |
| KPNA2 | karyopherin alpha 2 (RAG cohort 1, importin alpha 1) |
| KPNB1 | karyopherin (importin) beta 1 |
| KRAS | v-Ki-ras2 Kirsten rat sarcoma viral oncogene homolog |
| KRT8 | keratin 8 |
| LCN2 | lipocalin 2 |
| LRIG1 | leucine-rich repeats and immunoglobulin-like domains 1 |
| LRRN4 | leucine rich repeat neuronal 4 |
| MAL2 | mal, T-cell differentiation protein 2 |
| MALAT1 | metastasis associated lung adenocarcinoma transcript 1 (non-protein coding) |
| MAPK1 | mitogen-activated protein kinase 1 |
| MAPKSP1 | MAPK scaffold protein 1 |
| MDM4 | Mdm4 p53 binding protein homolog (mouse) |
| MGST1 | microsomal glutathione S-transferase 1 |
| MNDA | myeloid cell nuclear differentiation antigen |
| MSN | moesin |
| MTHFD2 | methylenetetrahydrofolate dehydrogenase (NADP+ dependent) 2, methenyltetrahydrofolate cyclohydrolase |
| MUC1 | mucin 1, cell surface associated |
| MUM1L1 | melanoma associated antigen (mutated) 1-like 1 |
| MYO1C | myosin IC |
| MYO1E | myosin IE |
| NAA50 | N(alpha)-acetyltransferase 50, NatE catalytic subunit |
| NCAPD2 | non-SMC condensin I complex, subunit D2 |
| NME1 | non-metastatic cells 1, protein (NM23A) expressed in |
| NRAS | neuroblastoma RAS viral (v-ras) oncogene homolog |
| NUAK2 | NUAK family, SNF1-like kinase, 2 |
| PAK1IP1 | PAK1 interacting protein 1 |
| PAK2 | p21 protein (Cdc42/Rac)-activated kinase 2 |
| PCOLCE2 | procollagen C-endopeptidase enhancer 2 |
| PDGFD | platelet derived growth factor D |
| PDIA6 | protein disulfide isomerase family A, member 6 |
| PFDN2 | prefoldin subunit 2 |
| PHF17 | PHD finger protein 17 |
| PRKCI | protein kinase C, iota |
| PSMD2 | proteasome (prosome, macropain) 26S subunit, non-ATPase, 2 |
| PTK2 | protein tyrosine kinase 2 |
| PTPRF | protein tyrosine phosphatase, receptor type, F |
| PTTG1 | pituitary tumor-transforming 1 |
| REEP1 | receptor accessory protein 1 |
| RPN1 | ribophorin I |
| S100A6 | S100 calcium binding protein A6 |
| SERP1 | stress-associated endoplasmic reticulum protein 1 |
| SERPINH1 | serpin peptidase inhibitor, clade H (heat shock protein 47), member 1, (collagen binding protein 1) |
| SFPQ | splicing factor proline/glutamine-rich |
| SLC34A2 | solute carrier family 34 (sodium phosphate), member 2 |
| SMARCA4 | SWI/SNF related, matrix associated, actin dependent regulator of chromatin, subfamily a, member 4 |
| SMC4 | structural maintenance of chromosomes 4 |
| SOCS3 | suppressor of cytokine signaling 3 |
| SPINT2 | serine peptidase inhibitor, Kunitz type, 2 |
| SPP1 | secreted phosphoprotein 1 |
| SPTAN1 | spectrin, alpha, non-erythrocytic 1 (alpha-fodrin) |
| SQLE | squalene epoxidase |
| ST13 | suppression of tumorigenicity 13 (colon carcinoma) (Hsp70 interacting protein) |
| STAT3 | signal transducer and activator of transcription 3 (acute-phase response factor) |
| STIP1 | stress-induced-phosphoprotein 1 |
| SUZ12 | suppressor of zeste 12 homolog (Drosophila) |
| TCEAL2 | transcription elongation factor A (SII)-like 2 |
| TEF | thyrotrophic embryonic factor |
| TIMP3 | TIMP metallopeptidase inhibitor 3 |
| TK2 | thymidine kinase 2, mitochondrial |
| TPD52 | tumor protein D52 |
| TPM4 | tropomyosin 4 |
| TTPAL | tocopherol (alpha) transfer protein-like |
| TUBA1B | tubulin, alpha 1b |
| TUBB | tubulin, beta |
| TUBB2C | tubulin, beta 2C |
| TUBGCP2 | tubulin, gamma complex associated protein 2 |
| UBE2C | ubiquitin-conjugating enzyme E2C |
| UBE4B | ubiquitination factor E4B (UFD2 homolog, yeast) |
| UGGT1 | UDP-glucose glycoprotein glucosyltransferase 1 |
| VEGFA | vascular endothelial growth factor A |
| WFDC2 | WAP four-disulfide core domain 2 |
| WNK4 | WNK lysine deficient protein kinase 4 |
| XBP1 | X-box binding protein 1 |
